# Noninvasive ventilation with helmet versus control strategy in patients with acute respiratory failure: a systematic review and meta-analysis of controlled studies

**DOI:** 10.1186/s13054-016-1449-4

**Published:** 2016-08-23

**Authors:** Qi Liu, Yonghua Gao, Rongchang Chen, Zhe Cheng

**Affiliations:** 1 Department of Pulmonary and Critical Care Medicine, the First Affiliated Hospital of Zhengzhou University, 1st Jianshe East Road, Zhengzhou, Henan 450001 People’s Republic of China; 2 Respiratory Mechanics Lab, State Key Laboratory of Respiratory Disease, Guangzhou Institute of Respiratory Disease, First Affiliated Hospital of Guangzhou Medical University, 151st Yanjiang West Road, Guangzhou, Guangdong 510120 People’s Republic of China

**Keywords:** Helmet, Noninvasive ventilation, Facial mask

## Abstract

**Background:**

Noninvasive ventilation (NIV) has proved to be a useful technique for breathing support. However, complications, discomfort, and failure of NIV were commonly caused by the mask. Therefore, the helmet was developed to improve performance and reduce complications; however, there has been no conclusive results on its effect until now. Thus, we performed a systematic review and meta-analysis to investigate the effect of NIV with a helmet versus the control strategy in patients with acute respiratory failure (ARF).

**Methods:**

We searched Cochrane Library, PubMed, Ovid, and Embase databases and bibliographies of relevant articles published before June 2016. Randomized and case-control studies that adopted the helmet as an NIV interface and compared it with another interface were included. The primary outcomes were hospital mortality, intubation rate, and complications. The secondary outcomes included the length of intensive care unit (ICU) stay, gas exchange, and respiratory rate. Pooled odds ratios (ORs) and 95 % confidence intervals (CIs) were calculated by the Mantel-Haenszel method and mean difference by the inverse variance method in a fixed effect model or random effects model according to the heterogeneity.

**Results:**

A total of 11 studies involving 621 patients were included. The overall hospital mortality was 17.53 % in the helmet NIV group versus 30.67 % in the control group. Use of the helmet was associated with lower hospital mortality (OR 0.43, 95 % CI 0.26 to 0.69, *p* = 0.0005), intubation rate (OR 0.32, 95 % CI 0.21 to 0.47, *P* < 0.00001), and complications (OR 0.6, 95 % CI 0.4 to 0.92, *P* = 0.02). In contrast, there was no significant difference in gas exchange and ICU stay (*P* >0.05). Subgroup analysis found the helmet reduced mortality mainly in hypoxemic ARF patients (*P* < 0.05) and a lower intubation rate was shown in randomized trials; fewer complications caused by the helmet might be restricted to case-control trials. Additionally, the effect of the helmet on PaCO_2_ was influenced by type of ARF and ventilation mode (*P* <0.00001).

**Conclusion:**

NIV with a helmet was associated with reduced hospital mortality and intubation requirement. The helmet was as effective as the mask in gas exchange with no additional advantage. Large randomized controlled trials are needed to provide more robust evidence.

## Background

Noninvasive ventilation (NIV) has proved to be a useful technique for breathing support that improved gas exchange and reduced the need for intubation and mortality in patients with exacerbation of chronic obstructive pulmonary disease, acute cardiogenic pulmonary edema, and blunt chest trauma [1–4]. However, the overall failure of NIV occurred in 16–30 % of patients [5, 6], which depended on not only the underlying cause of acute respiratory failure (ARF) and the severity of the patients’ disease, but also the multiple technical causes including interface-associated complications [7, 8].

The mask turned mechanical ventilation without intubation into reality and was a key success factor in NIV. Despite great improvements in the material quality, shape, size, and fixing system of the mask, complications such as skin lesions, nasal pain, ulcerations of the nose bridge, and discomfort were very common [7, 9, 10]. NIV might fail in 18 % of cases attributable to mask discomfort [11], and failure of facial mask-delivered NIV was associated with a threefold increase in hospital mortality [12]. In an attempt to improve performance and reduce complications, the helmet was developed, which was a new-fashioned interface with the advantage of avoiding skin lesions and improving patient comfort independent of face morphology [13, 14]. Nevertheless, there has been no clear consensus on its effect until now. Whether NIV delivered by helmet could reduce the intubation rate and mortality was still causing controversy [15, 16], furthermore, the effect on gas exchange was also confusing. Some studies indicated NIV delivered by helmet could improve oxygenation further compared with a face mask [17, 18], while others showed there was no significant difference in oxygenation [19–21], and some studies even demonstrated helmet-induced carbon dioxide rebreathing [22].

Therefore, we conducted a meta-analysis to investigate the effect of NIV with a helmet on hospital mortality, intubation rate, complications, and gas exchange versus the control strategy in patients with ARF.

## Methods

This systematic review and meta-analysis was performed in adherence to the recently published Preferred Reporting Items for Systematic Reviews and Meta-Analyses (PRISMA) statement [23] and our prespecified protocol. Ethical approval and patient consent were not required since this was a meta-analysis of previously published studies. The eligible participants were adults (at least 18 years old) who suffered from ARF defined by the widely accepted consensus and needed breathing support. Interventions in the experiment group were NIV with a standard or new-generation helmet independent of ventilation mode; breathing support strategies in the control group included NIV or oxygen therapy with any kind of noninvasive interface. The primary outcomes were hospital mortality, intubation rate, and complications. The secondary outcomes included the length of stay in the intensive care unit (ICU), arterial blood gas exchange [including pH, the ratio of arterial oxygen partial pressure to fractional inspired oxygen (PaO_2_/FiO_2_), and partial pressure of carbon dioxide in the blood (PaCO_2_)] and respiratory rate. Randomized and case-control trials that met the eligible criteria were included, while crossover studies were excluded.

### Search strategy

Two authors (QL and YG) performed a computerized search of the Cochrane Library, PubMed, Ovid, and Embase databases for articles published from inception to June 2016. The search strategy was a combination of keywords and terms as follows: helmet OR hood AND mechanical ventilation OR noninvasive ventilation OR oronasal mask OR facial mask OR nasal mask OR oxygen therapy. The searches were restricted to studies on humans without language limitations. The references of all included articles were checked manually to identify additional eligible studies. Duplicate articles were identified and deleted.

### Eligibility criteria

We screened for relevant studies that adopted the standard or new-generation helmet as an interface for breathing support. The inclusion criteria were as follow: (1) the main objective was to compare the effect of helmet ventilation with the control strategy, including randomized controlled trials (RCTs) and case-control studies; (2) adult patients with ARF; (3) outcomes were related to short-term effect such as gas exchange or long-term effect such as hospital mortality; (4) at least one outcome could be extracted. The exclusion criteria were: (1) helmet was introduced for prophylactic use; (2) trial subjects were postoperative patients, healthy volunteers, or simulators; (3) only a meeting paper or abstract was published without the full text; (4) studies without a control group; (5) editorials, case reports, letters, reviews, news, comments, guidelines, and meta-analyses. Checking of potentially eligible studies was performed independently by two authors (QL and YG), and in case of disagreement, both authors reviewed the article together until a consensus was made [24] and a third researcher (ZC) decided whether to include it or not.

### Data extraction and quality assessment

General material and outcomes were extracted from the selected articles. For dichotomous data (hospital mortality, intubation rate, and complications), number of events and patients in each group were picked up as the established protocol. For continuous data (gas exchange, respiratory rate, length of stay in ICU), we extracted the means, standard deviations, and the group sizes. The patient who refused intubation when meeting intubation criteria was included in the intubation group, the other data was extracted directly. To assess the possible risk of bias for RCTs, we used the Cochrane Collaboration tool, Table 8.5.a in the *Cochrane Handbook for Systematic Reviews of Interventions*, for assessing the risk of bias [25]. This tool covered six domains as follows: sequence generation, allocation concealment, blinding of participants and personnel, blinding of outcome assessment, incomplete outcome data, and selective outcome reporting. Each domain was rated as “high risk”, “low risk” or “unclear” of bias according to the relative information. To assess the possible risk of bias for case-control trials, we adopted the Newcastle-Ottawa scale (NOS), which focused on three categories: selection, comparability, and exposure with each being awarded a maximum of nine stars on items [26]. The judgements were made independently by two review authors (QL and YG). In case of disagreement, it was resolved first by discussion and then by consulting a third author (ZC) for arbitration.

### Statistical analysis

We performed our meta-analysis including all studies. For dichotomous outcomes, pooled odds ratio (OR) with 95 % confidence interval (CI) was estimated by the Mantel-Haenszel method. For continuous outcomes, pooled effect sizes were calculated by the inverse variance method and expressed as mean difference (MD) with 95 % CI. If the homogeneity across studies was sufficient, the analysis was performed using a fixed effect model, whereas if not, a random effects model was used. The heterogeneity across studies was tested by the I^2^ statistic, a quantitative measure of inconsistency [27, 28]. I^2^ values of 25–50 % indicated low, 50–70 % moderate, and >75 % high heterogeneity [24]. We further conducted subgroup analysis to explore possible explanations for heterogeneity. A *P* value of less than 0.05 was considered to be statistically significant for all analyses. Potential publication bias was assessed by visual inspection of the funnel plot in which the logarithms of ORs were plotted against their standard errors. Statistical analyses were performed with Review Manager 5.3 software (Cochrane Collaboration, Oxford, UK). We adopted Begg’s and Egger’s tests to evaluate the publication bias quantitatively and estimated further using a trim and fill method if necessary by STATA 12.0 (Stata Corp, College Station, TX, USA).

## Results

### Literature search and study identification

**Fig. 1:**
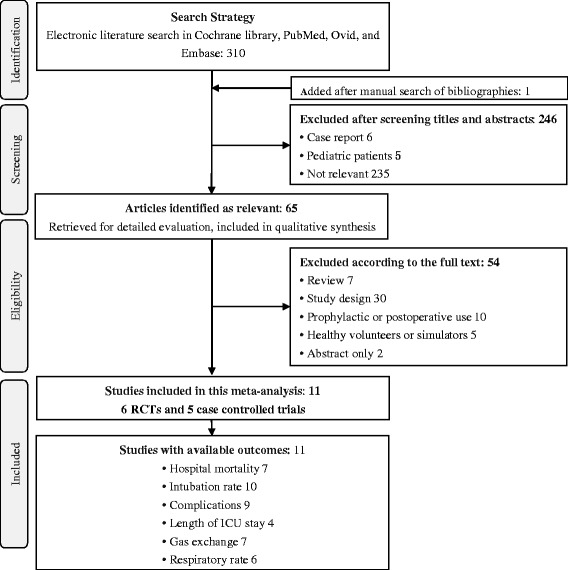
Flow chart showing the selection of studies in this meta-analysis

We found 310 articles according to the electronic search strategy and one additional study was added from the reference list of one article. The identifying process was shown in Fig. 1. A total of 246 studies were excluded according to the inclusion and exclusion criteria after screening titles and abstracts for relevance, patients recruited, and article type. Fifty-four studies were excluded after examination of the full text. Finally, 11 studies involving 621 patients, with six RCTs [15, 16, 29–32] and five case-control studies [14, 17, 20, 33, 34], were included for the meta-analysis. These studies recruited patients admitted to ICUs [14, 15, 20, 29–32, 34], emergency departments [33], high dependency units [16], and departments of hematology [17] in different countries: Italy [14–17, 20, 34], Turkey [29, 30], Brazil [31], America [32], and France [33]. The characteristics of the studies are shown in Table 1. Eight studies performed NIV through helmet with pressure support ventilation (PSV) [14, 15, 20, 29–32, 34] and three with continuous positive airway pressure (CPAP) [16, 17, 29]. In the control group, the interfaces were facial mask in nine studies [14, 17, 20, 29–34], oronasal mask [15] and Venturi mask [16] in one study, respectively.

**Table 1:** Characteristics of the included studies for helmet ventilation in the meta-analysis

Study	Study design	Patients	Experimental strategy	Control strategy	Sample size		Outcomes
					Helmet	Control	
Antonelli 2002 [14]	Prospective pilot case control	Patients with hypoxemic ARF without COPD	Helmet PSV	Facial mask PSV	33	66	Gas exchange, respiratory rate, intubation rate, length of NIV, level of pressure support, ICU stay and hospital mortality, complications
Pisani 2015 [15]	Multicenter RCT	AECOPD with hypercapnic ARF	Helmet PSV	Oronasal mask PSV	39	41	Respiratory rate, dysponea score, intubation rate, level of pressure support, hemodynamics, complications
Brambilla 2014 [16]	Multicenter RCT	Pneumonia with hypoxemic ARF	Helmet CPAP	Venturi mask Oxygen therapy	40	41	Gas exchange^a^, intubation rate, hospital stay and mortality, complications
Principi 2004 [17]	Historical case control	Hematological malignancy with hypoxemic ARF	Helmet CPAP	Facial mask CPAP	17	17	Gas exchange, intubation rate, PTS, length of NIV, complications
Antonelli 2004 [20]	Historical case control	AECOPD with hypercapnic ARF	Helmet PSV	Facial mask PSV	33	33	Gas exchange, respiratory rate, intubation rate, length of NIV, level of pressure support, hemodynamics, ICU stay, ICU and hospital mortality, complications
Ali 2011 [29]	RCT	AECOPD with hypercapnic ARF	Helmet PSV	Facial mask PSV	15	15	Gas exchange, respiratory rate, hemodynamics, ICU stay, PTS, intubation rate, complications
Özlem 2015 [30]	RCT	AECOPD with hypercapnic ARF	Helmet PSV	Facial mask PSV	25	23	Gas exchange^a^, respiratory rate^b^, PTS, complications, ICU stay^b^, length of NIV, hospital mortality
Antonaglia 2011 [31]	RCT	AECOPD with hypercapnic ARF	Helmet PSV	Facial mask PSV	20	20	Gas exchange, respiratory rate^b^, intolerance to the interface, length of ICU stay^b^, time of ventilator assistance
Patel 2016 [32]	RCT	ARF due to ARDS	Helmet PSV	Facial mask PSV	44	39	Respiratory rate^b^, intubation rate, length of NIV, level of pressure support, ICU and hospital stay^b^, hospital and 90d mortality, complications, ventilator-free days
Tonnelier 2003 [33]	Historical case control	Cardiogenic pulmonary edema with hypoxemic ARF	Helmet CPAP	Facial mask CPAP	11	11	Glasgow coma scale, gas exchange, respiratory rate, complications, hospital mortality
Rocco 2004 [34]	Historical case control	Immunocompromised with hypoxemic ARF	Helmet PSV	Facial mask PSV	19	19	Gas exchange, respiratory rate, intubation rate, length of NIV, level of pressure support, ICU stay, ICU and hospital mortality, complications

*ARF* acute respiratory failure, *COPD* chronic obstructive pulmonary disease, *PSV* pressure support ventilation, *NIV* noninvasive ventilation, *ICU* intensive care unit, *AECOPD* exacerbation of COPD, *CPAP* continuous positive airway pressure, *PTS* patient tolerance scale, *RCT* randomized control trail, *ARDS* acute respiratory distress syndrome

^a^Outcome presented in figure form

^b^Outcome presented as interquartile range

### Quality assessment

The results of quality assessment were shown in Table 2 and Table 3. As shown in Table 2, the scores of case-control trials ranged from five to eight stars, more than four stars were defined as high-quality. All studies reported the clear definition of cases and controls. All cases in each study were included during a certain period, in certain medical centers, and thus confirm the representativeness of cases. These items were awarded stars. Four historical controlled studies used different methods to ascertain exposure for cases and controls and the samples might be from different population because of inclusion from different periods [17, 20, 33, 34]. In the control group, one study with shorter NIV duration [17] and one with lower pressure support [20], we considered insufficient for additional control factors. No study mentioned the nonresponse rate. All of these conditions were not awarded stars. For the RCTs, as shown in Table 3, most of domains were evaluated as low risk of bias. Most notably, blinding of participants and personnel was not possible in these trials because of the nature of the intervention, i.e., NIV with a helmet or not, therefore performance bias was considered as high risk in all the studies. For the outcomes of interest, such as mortality, the outcome assessment was not affected by blinding or not, we evaluated the detection bias as low risk.

**Table 2:** Quality assessment of case-control studies included by NOS

Study, year	Selection				Comparability		Exposure			Overall stars
	Is the case definition adequate?	Representative of the cases	Selection of controls	Definition of controls	Control for important factors	Control for any additional factor	Ascertainment of exposure	Same method of ascertainment for cases and controls	Nonresponse rate	
Antonelli 2002 [14]	★	★	★	★	★	★	★	★	-	8
Principi 2004 [17]	★	★	-	★	★	-	★	-	-	5
Antonelli 2004 [20]	★	★	-	★	★	-	★	-	-	5
Tonnelier 2003 [33]	★	★	-	★	★	★	★	-	-	6
Rocco 2004 [34]	★	★	-	★	★	★	★	-	-	6

*NOS* Newcastle-Ottawa scale; ★ the quality met the criterion of this specific item, - the item was not qualified to be awarded a star

**Table 3:** Quality assessment of RCTs included by the Cochrane Collaboration tool

Study, year	Selection bias		Performance bias	Detection bias	Attrition bias	Reporting bias
	Random sequence generation	Allocation concealment	Blinding of participants and personnel	Blinding of outcome assessment	Incomplete outcome data assessments	Selective reporting.
Pisani 2015 [15]	Low	Low	High	Low	Low	Low
Brambilla 2014 [16]	Low	Low	High	Low	Low	Low
Ali 2011 [29]	Unclear	Unclear	High	High	Low	Low
Özlem 2015 [30]	Unclear	Unclear	High	High	Low	Low
Antonaglia 2011 [31]	Low	Low	High	High	Unclear	Low
Patel 2016 [32]	Low	Low	High	Low	Low	Low

*Low* low risk of bias, *High* high risk of bias, *Unclear* unclear risk of bias according to the relative information

### Effect of NIV with a helmet on hospital mortality

**Fig. 2:**
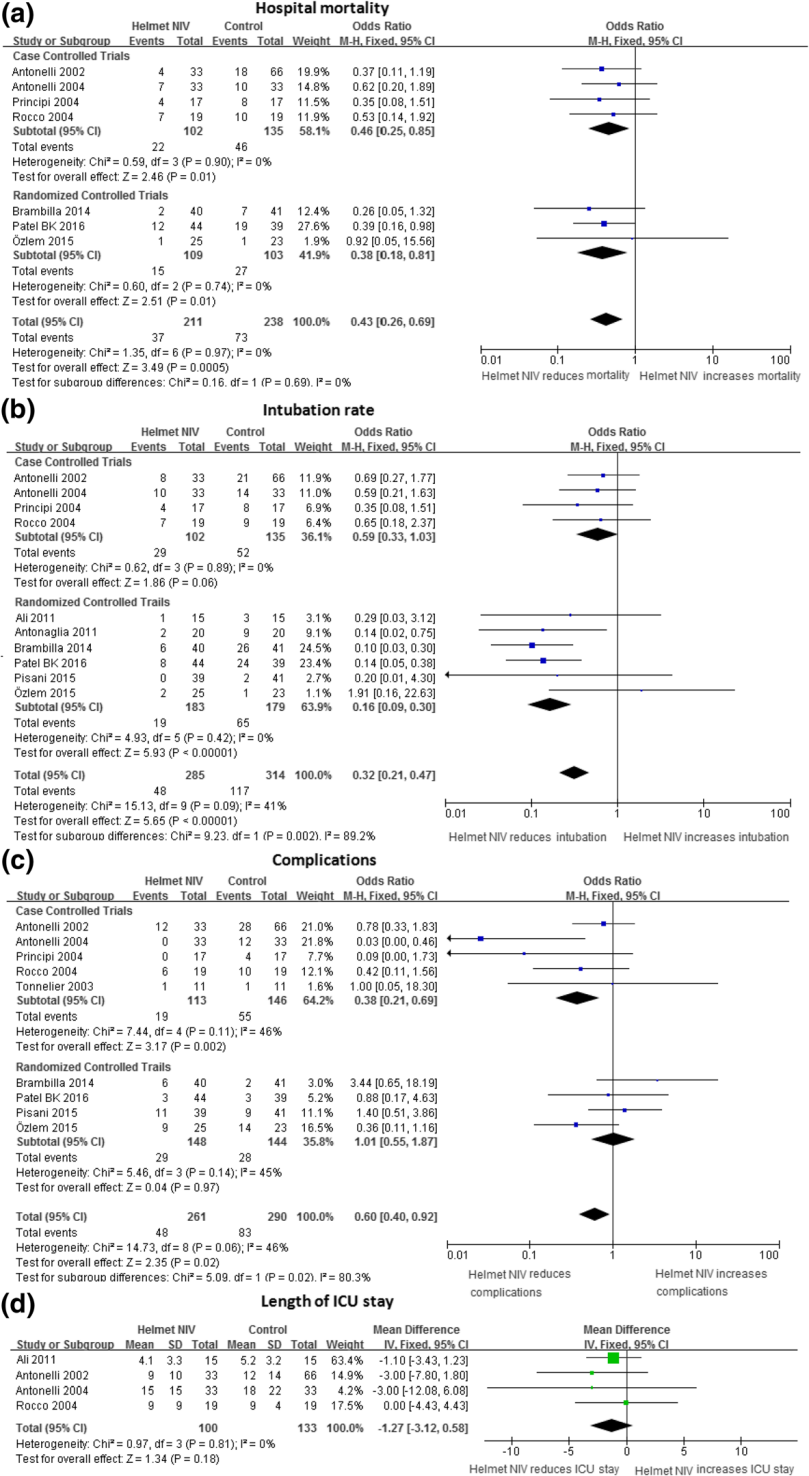
Effect of NIV with a helmet on primary outcomes and length of ICU stay. The helmet NIV group represents patients receiving NIV with a helmet, the control group represent patients receiving NIV with a mask or oxygen therapy with a mask. *Vertical solid line* null effect, *boxes* and *horizontal lines* outcome in the corresponding study and 95 % CI, *filled rhombic boxes* overall effect size. **a** Effect of NIV with a helmet on hospital mortality; **b** effect of NIV with a helmet on intubation rate; **c** effect of NIV with a helmet on complications; **d** effect of NIV with a helmet on length of ICU stay. *CI* confidence interval, *ICU* intensive care unit, *IV* inverse variance method, *NIV* noninvasive ventilation, *M-H* Mantel-Haenszel method, *SD* standard deviation

Seven studies including 449 patients reported hospital mortality. The pooled results are shown in Fig. 2a. The hospital mortality was 17.53 % (37/211) in the helmet NIV group compared to 30.67 % (73/238) in the control group. Pooled results showed the helmet was associated with lower hospital mortality (OR 0.43, 95 % CI 0.26 to 0.69, *P* = 0.0005). There was no heterogeneity found across studies within and between subgroups according to study design.

### Effect of NIV with a helmet on intubation rate, complications, and length of ICU stay

Ten studies recruiting 599 patients offered information about intubation. NIV with a helmet significantly reduced the intubation rate (16.85 % versus 37.26 %) and the OR was 0.32 with a 95 % CI from 0.21 to 0.47 (*P* < 0.00001), the heterogeneity was low with I^2^ 41 % (*P* = 0.09) (Fig. 2b). Overall complications in the helmet NIV group were less than the control group with OR 0.60 (95 % CI from 0.40 to 0.92, *P* = 0.02) (Fig. 2c). Only four articles expressed the data of length of ICU stay in the form of mean ± standard deviation. NIV with a helmet did not affect the length of ICU stay (MD −1.27, 95 % CI: −3.12 to 0.58, *P* = 0.18) (Fig. 2d).

### Effect of NIV with a helmet on gas exchange and respiratory rate

**Fig. 3:**
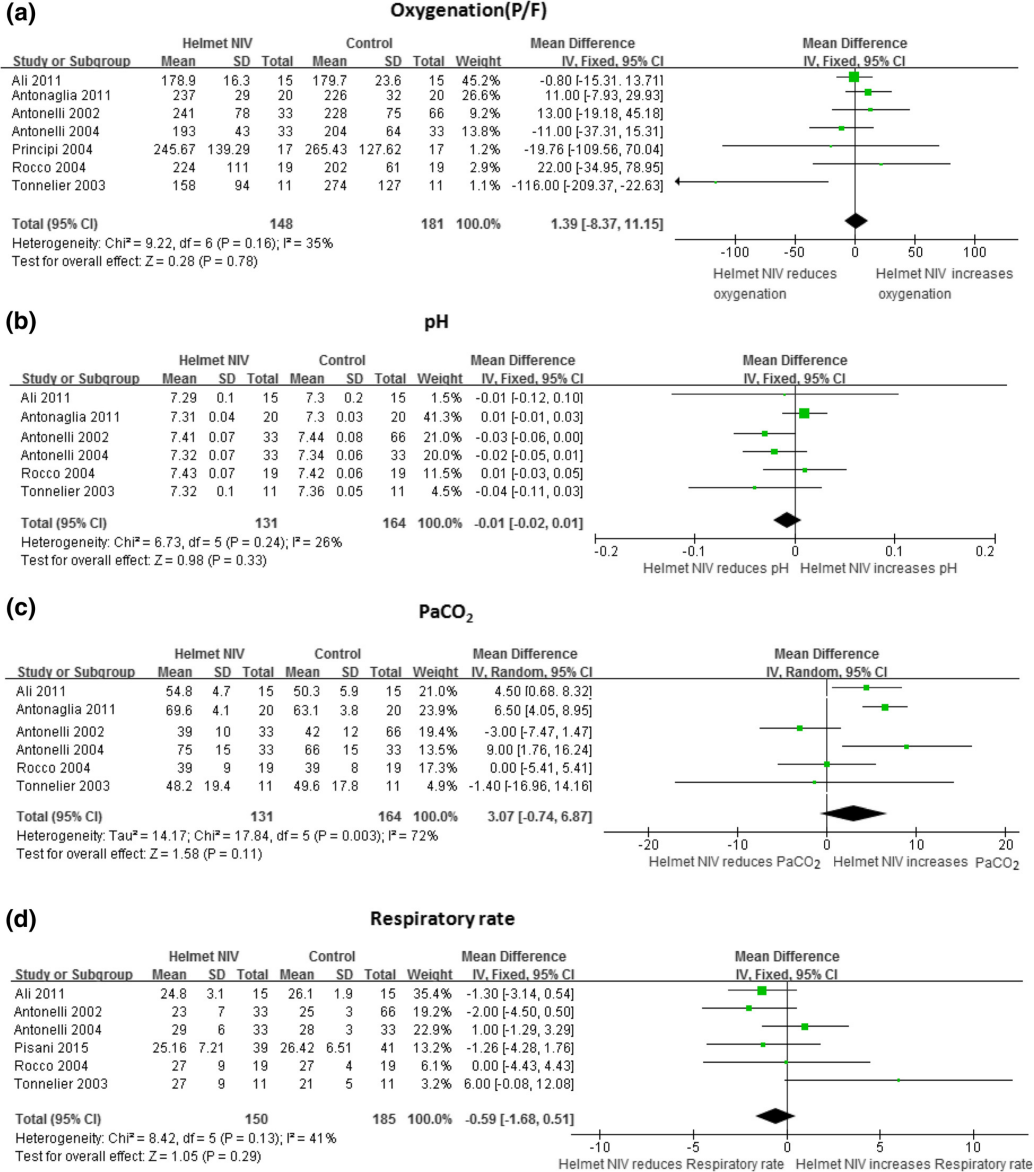
Effect of NIV with a helmet on the gas exchange and respiratory rate. The helmet NIV group represents patients receiving NIV with a helmet, the control group represent patients receiving NIV with mask or oxygen therapy with mask. *Vertical solid line* null effect, *boxes* and *horizontal lines* outcome in the corresponding study and 95 % CI, *filled rhombic boxes* overall effect size. **a** Effect of NIV with a helmet on oxygenation; **b** effect of NIV a with helmet on pH; **c** effect of NIV with a helmet on PaCO_2_; **d** effect of NIV with a helmet on respiratory rate. *CI* confidence interval, *IV* inverse variance method, *NIV* noninvasive ventilation, *PaCO*_*2*_ partial pressure of carbon dioxide in arterial blood, *P/F* the ratio of partial pressure of oxygenation in arterial blood to fraction of inspired oxygenation, *SD* standard deviation,

NIV with a helmet had the same effect on the improvement of PaO_2_/FiO_2_ and arterial pH as the control strategy (*P* > 0.05) (Fig. 3a, b). PaCO_2_ in the helmet NIV group was not higher than the control group, but the heterogeneity was not perfect with I^2^ equal to 72 % (*P* = 0.11) (Fig. 3c). The respiratory rate was similar in both groups and MD was −0.59 (95 % CI: −1.68 to 0.51, *P* = 0.29) (Fig. 3d).

### Subgroup analysis

Trials both designed in randomization and case-control, showed use of a helmet was associated with lower hospitality mortality (Fig. 2). As shown in Table 4, further subgroup analysis according to the type of ARF found that NIV with a helmet reduced mortality mainly in the patient group with hypoxemic ARF with OR 0.38 (0.22, 0.65) (*P* = 0.0005), and helmet with both CPAP and PSV could reduce mortality (*P* < 0.05). NIV with a helmet could decrease the intubation in both hypercapnic and hypoxemic ARF patients independent of the ventilation mode (*P* < 0.05). Fewer complications occurred in the subgroup patients with hypercapnic ARF (*P* < 0.05). PaCO_2_ could be decreased further in hypercapnic patients or ventilation with PSV (*P* < 0.00001), the effect of a helmet on the continuous outcomes (the ICU stay, oxygenation, pH, and respiratory rate) were not influenced by type of ARF and ventilation mode, a helmet improved pH mainly shown by case-control trials (*P* = 0.04) (Table 5), in addition interfaces used in the control group did not affect the final result significantly (Tables 4 and 5).

**Table 4:** Subgroup analysis - the effect on primary outcomes

	No. of studies	Helmet NIV event/total	Control event/total	I^2^ (%)	*P* value for heterogeneity	OR 95 % CI	Overall effect Z/*P* value
Hospital mortality							
Type of ARF							
Hypercapnic	2	8/58	11/56	0	0.8	0.65 [0.28, 2.06]	0.81/0.42
Hypoxemic	5	29/153	62/182	0	0.97	0.38 [0.22, 0.65]	3.49/0.0005
Ventilation mode in the experimental group							
CPAP	2	6/57	15/58	0	0.79	0.30 [0.10, 0.89]	2.17/0.03
PSV	5	31/154	58/180	0	0.77	0.46 [0.27, 0.79]	2.8/0.005
Interfaces used in the control group							
Facial mask	6	35/171	66/197	0	0.97	0.45 [0.27, 0.74]	3.12/0.002
Venturi mask	1	2/40	7/41	NA	NA	0.26 [0.05, 1.32]	1.63/0.1
Intubation rate							
Type of ARF							
Hypercapnic	5	15/132	29/132	0	0.42	0.42 [0.20, 0.85]	2.41/0.02
Hypoxemic	5	33/153	88/143	61	0.03	0.28 [0.17, 0.45]	5.16/<0.0001
Ventilation mode in the experimental group							
CPAP	2	10/57	34/58	42	0.19	0.16 [0.07, 0.36]	4.28/<0.0001
PSV	8	38/228	83/256	30	0.19	0.39 [0.25, 0.62]	4.05/<0.0001
Interfaces used in the control group							
Facial mask	8	42/206	89/232	29	0.2	0.39 [0.25, 0.61]	4.16/<0.0001
Oronasal mask	1	0/39	2/41	NA	NA	0.20 [0.01, 4.30]	1.03/0.3
Venturi mask	1	6/40	26/41	NA	NA	0.32 [0.21, 0.47]	5.56/<0.0001
Complications							
Type of ARF							
Hypercapnic	3	20/97	35/97	77	0.01	0.45 [0.23, 0.86]	2.42/0.02
Hypoxemic	6	28/164	48/193	17	0.30	0.76 [0.43, 1.32]	0.98/0.33
Ventilation mode in the experimental group							
CPAP	3	7/68	7/69	57	0.10	1.01 [0.36, 2.89]	0.03/0.98
PSV	6	41/193	76/221	46	0.10	0.55 [0.34, 0.87]	2.35/0.06
Interfaces used in the control group							
Facial mask	7	31/182	72/208	25	0.24	0.40 [0.24, 0.67]	3.49/0.0005
Oronasal mask	1	11/39	9/41	NA	NA	1.40 [0.51, 3.86]	0.64/0. 52
Venturi mask	1	6/40	2/41	NA	NA	3.44 [0.65, 18.2]	1.45/0.15

*NIV* noninvasive ventilation, *OR* odds ratio, *CI* confidence interval, *ARF* acute respiratory failure, *CPAP* continuous positive airway pressure, *PSV* pressure support ventilation, *NA* not applicable

**Table 5:** Subgroup analysis - the effect on secondary outcomes

	No. of studies	Patients (n) H/C	I^2^ (%)	*P* value for heterogeneity	MD 95 % CI	Overall effect Z/*P* value
Length of ICU stay						
Type of ARF						
Hypercapnic	2	48/48	0	0.69	−1.22 [−3.47, 1.04]	1.06/0.29
Hypoxemic	2	52/85	0	0.37	−1.38 [−4.63, 1.88]	0.83/0.41
Ventilation mode in the experimental group						
CPAP	1	15/15	NA	NA	−1.10 [−3.43, 1.23]	0.93/0.35
PSV	3	85/118	0	0.63	−1.56 [−4.63, 1.50]	1/0.32
Study design						
Case-control trial	3	85/118	0	0.63	−1.56 [−4.63, 1.50]	1/0.32
RCT	1	15/15	NA	NA	−1.10 [−3.43, 1.23]	0.93/0.35
Oxygenation (PaO_2_/FiO_2_)						
Type of ARF						
Hypercapnic	3	68/68	0	0.38	1.23 [−9.32, 11.78]	0.23/0.38
Hypoxemic	4	80/113	59	0.06	2.37 [−23.34, 28.08]	0.18/0.86
Ventilation mode in the experimental group						
CPAP	2	28/28	53	0.15	−66.01 [−130.73, −1.28]	2/0.05
PSV	5	120/153	0	0.59	2.96 [−6.91, 12.83]	0.59/0.56
Study design						
Case-control trial	5	113/146	49	0.1	4.16 [−22.55, 14.22]	0.44/0.66
RCT	2	35/35	0	0.33	3.57 [−7.95, 15.09]	0.61/0.54
pH						
Type of ARF						
Hypercapnic	3	68/68	16	0.3	−0.00 [−0.02, 0.02]	0.01/1
Hypoxemic	3	63/96	28	0.25	−0.02 [−0.04, 0.00]	1.59/0.11
Ventilation mode in the experimental group						
CPAP	1	11/11	NA	NA	−0.04 [−0.11, 0.03]	1.19/0.24
PSV	5	120/153	30	0.22	−0.01 [−0.02, 0.01]	0.74/0.46
Study design						
Case-control trial	4	96/129	0	0.43	−0.02 [−0.04, −0.00]	2.02/0.04
RCT	2	35/35	0	0.73	0.01 [−0.01, 0.03]	0.85/0.4
PaCO_2_						
Type of ARF						
Hypercapnic	3	68/68	0	0.5	6.15 [4.17, 8.13]	6.08/<0.00001
Hypoxemic	3	63/96	0	0.7	−1.76 [−5.13, 1.60]	1.03/0.30
Ventilation mode in the experimental group						
CPAP	1	11/11	NA	NA	−1.40 [−16.96, 14.16]	0.18/0.86
PSV	5	120/153	77	0.002	4.18 [2.46, 5.90]	4.76/<0.00001
Study design						
Case-control trial	4	96/129	61	0.05	0.15 [−2.90, 3.20]	0.1/<0.92
RCT	2	35/35	0	0.39	5.92 [3.85, 7.98]	5.62/<0.00001
Respiratory rate						
Type of ARF						
Hypercapnic	3	87/89	23	0.27	−0.56 [−1.85, 0.74]	0.84/0.4
Hypoxemic	3	63/96	65	0.06	0.58 [−3.52, 4.69]	0.28/0.78
Ventilation mode in the experimental group						
CPAP	1	11/11	NA	NA	6.00 [−0.08, 12.08]	1.93/0.05
PSV	5	139/174	0	0.44	−0.81 [−1.92, 0.30]	1.42/0.15
Study design						
Case-control trial	4	96/129	57	0.07	0.07 [−1.45, 1.60]	0.1/0.92
RCT	2	54/56	0	0.98	−1.29 [−2.86, 0.28]	1.61/0.11
Interfaces used in the control group						
Facial mask	5	111/144	51	0.08	−0.49 [−1.66, 0.69]	0.81/0.42
Oronasal mask	1	39/41	NA	NA	−1.26 [−4.28, 1.76]	0.82/0.41

*H/C* helmet/control, *MD* mean difference, *CI* confidence interval, *ICU* intensive care unit, *ARF* acute respiratory failure, *CPAP* continuous positive airway pressure, *PSV* pressure support ventilation, high quality randomized controlled trial, *PaO*_*2*_ */FiO*_*2*_ the ratio of the partial pressure of oxygen in arterial blood to the inspired oxygen fraction, *pH* potential of hydrogen, *PaCO*_*2*_ partial pressure of carbon dioxide, *NA* not applicable

### Publication bias

**Fig. 4:**
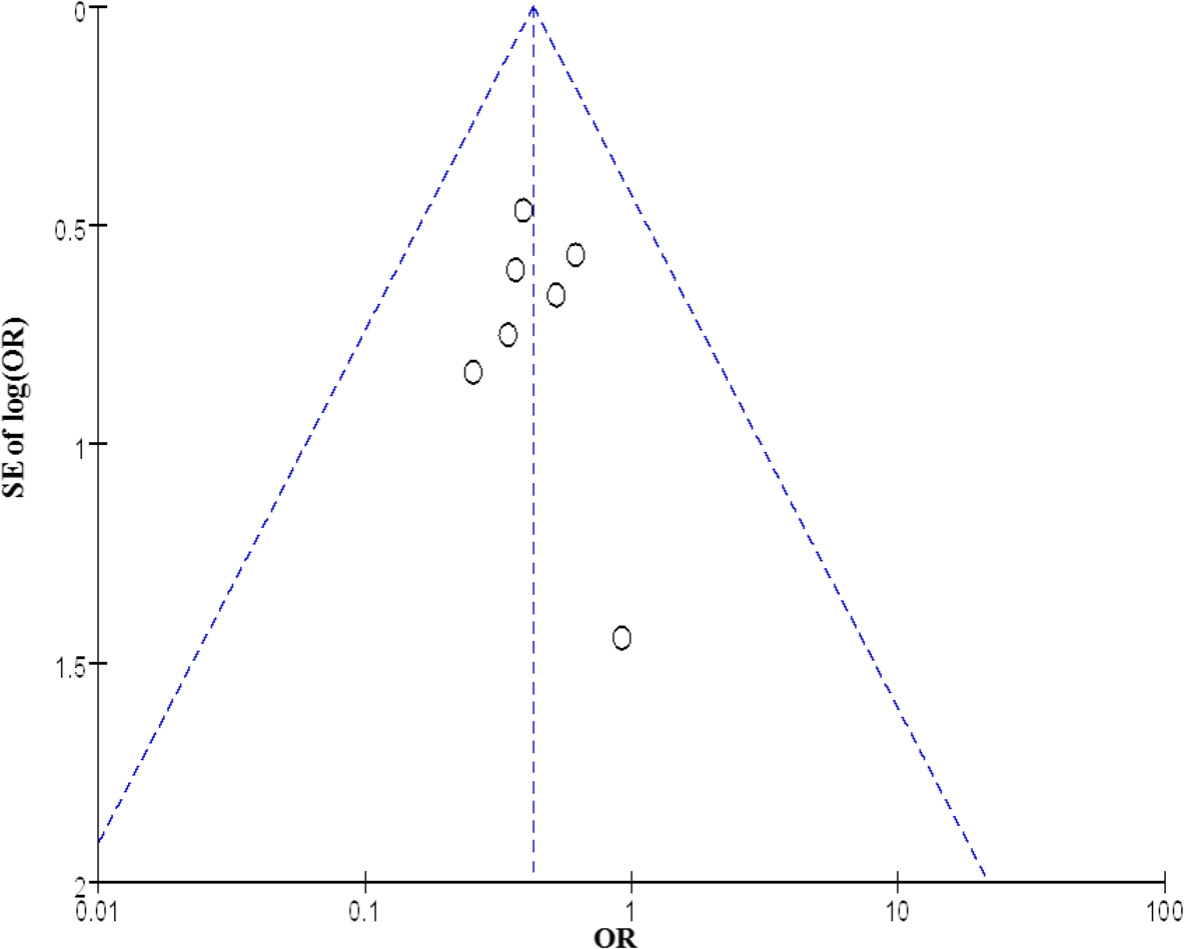
Funnel plots to assess publication bias regarding the effect of NIV with a helmet on hospital mortality. *SE* standard error, *OR* odds ratio

Visual inspection of the funnel plot indicated a little asymmetry for the effect of NIV with a helmet on hospital mortality (Fig. 4). The publication bias was significant when estimated by Egger’s test (*P* = 0.014) and was not consistent when estimated by Begg’s test (*P* = 0.072). Nevertheless, the pooled results, tested further via trim and fill method, were credible and steady.

## Discussion

To our knowledge, this is the first meta-analysis to investigate the effect of NIV with a helmet on ARF patients. We found that NIV with a helmet significantly reduced hospital mortality, tracheal intubation rate, and complications compared with the control techniques. And subgroup analyses found NIV with a helmet, especially in PSV mode, could decrease the PaCO_2_ in hypercapnic patients, but did not increase PaCO_2_ in the overall group. The helmet was as efficient as a mask on shortening the length of ICU stay, improving oxygenation and pH.

NIV delivered breathing support without the use of endotracheal or tracheostomy tube. The interface between the patients and NIV ventilators included nasal, oronasal, and facial mask. These noninvasive interfaces differentiated NIV from invasive mechanical ventilation, and maintained the airway protection mechanisms and the patient’s ability to swallow. Well- cooperated NIV decreased mortality in selected patients [1, 2, 35–37]. However, patient discomfort and complications might limit continuous application of NIV [7], further decrease its efficacy, and even increase mortality. Some of these complications and discomfort were related to the mask [7–9, 38]. In order to improve tolerability for patients, a transparent helmet made from latex-free polyvinyl chloride was developed [13].

This meta-analysis has shown NIV with a helmet reduced hospital mortality further compared with other masks in both RCTs and case-control trials, which meant the helmet itself had an effect on the patient’s survival. This effect might be attributed to the unique advantages that masks did not possess. First, the helmet allowed patients to freely drink, communicate and expectorate. It improved clearance of sputum and collaboration with caregivers without interference of the NIV. Second, the helmet was a better tolerated interface, patients need not try hard to cater to the interface as with a mask. The patient tolerance scale in the helmet group was remarkably higher than in the facial mask control group [14, 17, 29, 39]. Better tolerance allowed a longer continuous application [14, 31] and increased the success rate of NIV [40]. In fact, no patients failed NIV as a result of claustrophobia, discomfort, or pain related to a helmet while 38 % patients might have a failed NIV owing to a mask [14], which would increase hospital mortality. Third, the helmet could be applied to any patient regardless of their face contour. During the course of NIV with a mask, 24–64 % of patients had to change to another type of mask [12, 41] and the most common reason for non-adaptation to the mask was the shape of the face [41, 42]. Unlike the mask, the helmet could also be used in difficult anatomical situations such as in edentulous or facial trauma patients [13]. In short, good amenity, better tolerance, and universal application of the helmet ensured NIV was better used and reduced intubation (as shown in this analysis in Fig. 2b), which might contribute to preventing ventilator-associated pneumonia and decreasing hospital mortality [43].

An exciting finding was that the helmet could reduce the mortality of patients with hypoxemic ARF. Some studies aimed to explore applying NIV through a mask in this kind of patient, however, the results were disappointing, successful treatment was lower with a limited effect on the prognosis [43–46]. This might be relevant to the different breathing patterns in hypoxemic ARF and hypercapnic ARF patients. In patients with hypoxemic ARF, mouth breathing was more common, with unavoidable air leaks although a facial or whole face mask was used [47]. The helmet improved tolerance and increased efficient ventilation, which was very important for patients with hypoxemic ARF. The continuous application of NIV was crucial in the onset phases of ARF, which otherwise might forebode NIV failure and life-threatening consequences [14, 48–50]. It should be noted that this finding did not mean NIV with a helmet had no effect on the mortality of patients with hypercapnic ARF, since only two studies with small sample sizes had reported this variable in this meta-analysis [20, 30]; this question warrant further investigation.

The pooled effect indicated that NIV through a helmet decreased complications and intubation rate dramatically. The helmet was a transparent plastic hood, which did not come into contact with the patient’s face, especially the nose bridge and did not cause skin lesions [13]. With this device, air leaks were localized around the neck, which prevented eye irritation and conjunctivitis. The fixation system was not as complicated as the traditional mask. A simplified fixation system should carry a lower risk of cutaneous lesions. The new-generation helmet, characterized by an annular openable ring placed underneath an inflatable cushion, further reduced discomfort and axillary skin lesions caused by padded armpit braces in a standard helmet, so complications might only be confined to the neck [51]. Fewer complications aided the patients’ adherence to the NIV and increased the success rate although the effect on reducing complications was mainly observed in observational studies and larger RCTs are still needed to confirm the results. In addition, the use of specific ventilator settings with the fastest pressurization rate, higher inspiratory and expiratory pressures could improve patient ventilator interaction [52, 53]. All of these key issues prevented some patients from intubation.

Our analysis informed that NIV via a helmet was as effective as existing masks in gas exchange although respiratory rates, reflecting the degree of dyspnea to a certain extent, did not decrease noticeably. NIV with a helmet could improve oxygenation by delivering high oxygen concentration, unloading respiratory muscles, recruiting alveoli and increasing functional lung volumes, which was no different from NIV with other interfaces or invasive mechanical ventilation [54]. Moreover, the helmet further reduced leaks in contrast with masks. After all, the aim of introducing the helmet, still a noninvasive interface, was to reduce complications and increase patient comfort, to achieve better use of NIV and improve the prognosis, not just to improve gas exchange further. In theory, the helmet predisposed to CO_2_ rebreathing, meanwhile the CO_2_ concentration within the interface depended on the patient’s CO_2_ production and could be minimized by specific settings including high fresh gas flow or higher inspiratory pressure [55–57]. NIV through a helmet might take a longer time to achieve the target level of pressure support due to its larger inner volume. At a lower level of pressure support, the helmet might not decrease the work of breathing and has a restricted effect on respiratory rate.

In the past three decades, NIV played a critical role in the treatment of ARF, especially using CPAP and PSV. From a physiological rationale level, PSV could provide more benefit than CPAP. Regrettably, PSV has not been found to offer any advantage in terms of intubation or mortality [58]. As shown in the subgroup analysis by ventilation mode, both CPAP and PSV with a helmet could reduce the mortality and intubation rate, although PaCO_2_ decreased mainly in the subgroup with PSV, and improve prognosis. This result was similar with the findings in patients with cardiogenic pulmonary edema [58].

Several limitations should be considered when interpreting our findings. First, because of the paucity of studies in this novel strategy, both RCTs and case-control trials were included. Fortunately, the results were substantially confirmed by pooled RCTs or case-control trials separately with low heterogeneity among the studies. Second, patients recruited in the included studies suffered from hypercapnic or hypoxemic ARF and were not highly consistent with each other. Third, the ventilation characteristics were not homogeneous, some were in CPAP mode, others in PSV, and the interface in the control group involved facial, nasal, oronasal and Venturi masks. Fourth, high heterogeneity existed across the studies for the variable PaCO_2_ (I^2^ equal to 72 %), this could be partly explained by the ARF type. In addition, publication bias might exist measuring by Begg’s and Egger’s test, but the final results were credible and steady tested by the trim and fill method, which tend to be more efficient when limited studies are included in a meta-analysis [59]. Finally, all of the included RCTs had high risk of performance bias attributed to the dramatic difference between a helmet and mask, which made it impossible for participants and personnel blinding to the interface and might influence the outcomes in a certain extent, we suggest that more attention should be paid to this key issue in future researches.

## Conclusions

In conclusion, this meta-analysis indicated NIV with a helmet improved the prognosis, reduced the requirement of intubation and complications. The helmet was as effective as the mask in gas exchange despite no additional advantage. It should be noted that there is not sufficient scientific evidence to recommend it in designated patients due to the limited number of trials available. Large RCT studies are still needed to provide more robust evidence.

## Key messages

- NIV with a helmet improved the prognosis, reduced the requirement of intubation and complications. The helmet was as effective as the mask in gas exchange although without any additional advantage.

## Abbreviations

ARF: Acute respiratory failure
CI: Confidence interval
ICU: Intensive care unit
MD: Mean difference
NIV: Noninvasive ventilation
OR: Odds ratio
PaCO_2_: Partial pressure of carbon dioxide in arterial blood
PaO_2_/FiO_2_: The ratio of the partial pressure of oxygen in arterial blood to the inspired oxygen fraction
PSV: Pressure support ventilation
RCT: Randomized controlled trial
SD: Standard deviation
